# Molecular genetics of idiopathic pulmonary fibrosis

**DOI:** 10.18699/VJGB-22-37

**Published:** 2022-05

**Authors:** R.N. Mustafin

**Affiliations:** Bashkir State Medical University, Ufa, Russia

**Keywords:** idiopathic pulmonary fibrosis, immune system, microRNA, telomeres, transposons, epigenetic factors, идиопатический легочный фиброз, иммунная система, микроРНК, теломеры, транспозоны, эпигенетические факторы

## Abstract

Idiopathic pulmonary fibrosis (IPF) is a severe progressive interstitial lung disease with a prevalence of 2 to 29 per 100,000 of the world’s population. Aging is a significant risk factor for IPF, and the mechanisms of aging (telomere depletion, genomic instability, mitochondrial dysfunction, loss of proteostasis) are involved in the pathogenesis of IPF. The pathogenesis of IPF consists of TGF-β activation, epithelial-mesenchymal transition, and SIRT7 expression decrease. Genetic studies have shown a role of mutations and polymorphisms in mucin genes (MUC5B), in the genes responsible for the integrity of telomeres (TERC, TERC, TINF2, DKC1, RTEL1, PARN), in surfactant-related genes (SFTPC, SFTPCA, SFTPA2, ABCA3, SP-A2), immune system genes (IL1RN, TOLLIP), and haplotypes of HLA genes (DRB1*15:01, DQB1*06:02) in IPF pathogenesis. The investigation of the influence of reversible epigenetic factors on the development of the disease, which can be corrected by targeted therapy, shows promise. Among them, an association of a number of specific microRNAs and long noncoding RNAs was revealed with IPF. Therefore, dysregulation of transposons, which serve as key sources of noncoding RNA and affect mechanisms of aging, may serve as a driver for IPF development. This is due to the fact that pathological activation of transposons leads to violation of the regulation of genes, in the epigenetic control of which microRNA originating from these transposons are involved (due to the complementarity of nucleotide sequences). Analysis of the MDTE database (miRNAs derived from Transposable Elements) allowed the detection of 12 different miRNAs derived in evolution from transposons and associated with IPF (miR-31, miR-302, miR-326, miR-335, miR-340, miR-374, miR-487, miR-493, miR-495, miR-630, miR-708, miR-1343). We described the relationship of transposons with TGF-β, sirtuins and telomeres, dysfunction of which is involved in the pathogenesis of IPF. New data on IPF epigenetic mechanisms can become the basis for improving results of targeted therapy of the disease using noncoding RNAs.

## Abstract (ru)

Идиопатический легочный фиброз – тяжелая прогрессирующая интерстициальная болезнь легких с распространенностью 2–29 случаев на 100 000 человек населения в мире. Значимым фактором риска заболевания является старение, механизмы развития которого задействованы в патогенезе идиопатического легочного фиброза. К ним относятся истощение теломер, геномная нестабильность, дисфункция митохондрий и потеря протеостаза. Важную роль в развитии идиопатического легочного фиброза играют также эпителиально-мезенхимальный переход, активация TGF-β и снижение экспрессии сиртуина SIRT7. Молекулярно-генетические исследования показали, что в патогенезе идиопатического легочного фиброза имеют значение мутации и полиморфизмы в генах муцина (MUC5B), в генах, ответственных за целостность теломер (TERC, TERT, TINF2, DKC1, RTEL1, PARN), генов сурфактанта (SFTPC, SFTPCA, SFTPA2, ABCA3, SP-A2) и иммунной системы (IL1RN, TOLLIP), а также гаплотипы генов HLA (DRB1*15:01, DQB1*06:02). Перспективно изучение влияния на развитие болезни обратимых эпигенетических факторов, которые могут быть скорректированы таргетной терапией. Среди них с идиопатическим легочным фиброзом ассоциированы специфические микроРНК и длинные некодирующие РНК. Сделано предположение, что драйверным событием для идиопатического легочного фиброза служит дисрегуляция транспозонов, которые являются ключевыми источниками некодирующих РНК и влияют на механизмы старения. Это обусловлено тем, что при патологической активации транспозонов происходит нарушение регуляции генов, в эпигенетическом управлении которых участвуют происходящие от этих транспозонов микроРНК (в связи с комплементарностью нуклеотидных последовательностей). Анализ базы данных MDTE (miRNAs derived from Transposable Elements) позволил выявить 12 различных микроРНК, гены которых в эволюции возникли от транспозонов и ассоциированы с идиопатическим легочным фиброзом (miR-31, miR-302, miR-326, miR-335, miR-340, miR-374, miR-487, miR-493, miR-495, miR-630, miR-708, miR-1343). Описаны взаимосвязи мобильных элементов с TGF-β, сиртуинами и теломерами, дисфункция которых вовлечена в патогенез идиопатического легочного фиброза. Новые данные об эпигенетических механизмах развития патологии могут стать основой для улучшения результатов таргетной терапии болезни с использованием в качестве мишени некодирующих РНК.

## Introduction

Idiopathic pulmonary fibrosis (IPF) is a progressive severe interstitial lung disease. The annual incidence of IPF is up to 17.4 per 100,000 people in the world (Chioma, Drake, 2017). The prevalence of IPF in different countries ranges from 2 to 29 per 100,000 people (Zhao et al., 2017) (for example, in Finland – 16–18 per 100,000 (Hodgson et al., 2002); in the USA – 14–42,7 per 100,000 people). IPF is associated with aging. Therefore, for people over 75 years of age, the prevalence of the disease is 227.2 per 100,000, while for people aged 18 to 34 years, the prevalence of IPF is 4 per 100,000. The average age of patients with IPF is 66 years (Raghu et al., 2006). Survival for IPF is about 3 years after diagnosis, and available drugs only slow the decline in lung function with little to no effect on mortality (Wyman et al., 2017).

IPF pathogenesis involves environmental influences and microorganisms (Sgalla et al., 2018). Viral (Epstein–Barr, cytomegalovirus, herpesvirus-1,-7,-8, Kaposi’s sarcoma and hepatitis C), bacterial and fungal infections play a potential role in the development of IPF (Sheng et al., 2020). Smoking and metal dust inhalation are also associated with the risk of IPF (Chioma, Drake, 2017; Sgalla et al., 2018). The development of IPF is affected by occupational hazards, such as contact with silicon, beryllium, coal dust, asbestos, and radiation. In addition, IPF is associated with anti-inflammatory drugs (sulfasalazine, rituximab), chemotherapy drugs (bleomycin, methotrexate), heart drugs (amiodarone, propranolol), and antibiotics (nitrofurantoin, ethambutol) (Chioma, Drake, 2017). In 2019, a meta-analysis including 3206 patients and 9368 healthy individuals showed the role of gastroesophageal reflux disease in the development of IPF (Methot et al., 2019).

According to the generally accepted hypothesis, IPF develops as a result of immune reactions to restore the structure of lung tissue in case of repeated damage to the alveolar epithelium or endothelium. In this mechanism, the inflammatory mediator profibrotic cytokine – transforming growth factor β (TGF-β) activates angiogenesis and the production of extracellular matrix components (collagen and fibronectin). Failure to inactivate the fibrotic trigger leads to an exacerbation of the inflammatory response with excessive deposition of matrix components and lung scarring (Chioma, Drake, 2017). Molecular mediators of IPF include cell surface proteins, intracellular proteins, and soluble molecules (cytokines). The development of IPF is associated with sirtuins, a family of histone deacetylases that require NAD+ for their catalytic activity. The expression of sirtuins in fibroblasts of patients with IPF is significantly reduced. Similarly, a decrease in the concentration of SIRT7 in lung tissues was found in experimental mouse models with IPF induced by bleomycin. Inhibition of SIRT7 in fibroblast cultures by siRNA caused an increase in collagen synthesis. Overexpression of SIRT7 in lung fibroblasts leads to lower levels of COL1A1, COL1A2, COL3A1, exerting an antifibrotic effect (Wyman et al., 2017).

In the pathogenesis of IPF, an important role is played by the epithelial-mesenchymal transition, during which the expression of adhesion molecules (E-cadherin) is suppressed, and the cytokeratin cytoskeleton is transformed into a vimentin one. Accordingly, epithelial cells acquire a mesenchymal morphology (Li J. et al., 2021). However, there is still no complete theory that would fully explain the mechanism of IPF development. The most accurate data on the pathogenesis of IPF can be obtained using molecular genetic studies, which are promising for identifying the individual risk of the disease and developing its effective targeted therapy (Spagnolo, Cottin, 2017).

## Genetic factors in idiopathic pulmonary fibrosis

Familial IPF involving two or more family members averages 10 to 15 % of all IPF cases (Chioma, Drake, 2017). There are sporadic, familial and syndromal forms of IPF (Lawson et al., 2004; Gochuico et al., 2012). Sporadic cases of the disease are multifactorial diseases, that is, their development is influenced by environmental factors. These forms comprise the majority of IPF cases and are associated with polymorphic variants of various genes (Table 1). Risk factors for sporadic IPFs are male gender, smoking, inhalation of metal and wood dust, or use of certain medications such as methotrexate and bleomycin (Fernandez et al., 2012). Familial IPFs are similar to sporadic, but are characterized by an earlier manifestation. They are caused by mutations in certain genes (see Table 1) (Lawson et al., 2004).

**Table 1.**
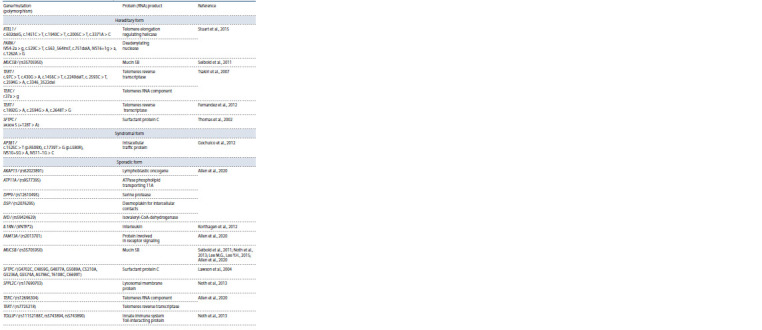
Genetics of various forms of idiopathic pulmonary fibrosis

Familial IPFs were first described in 1958 by McKusick and Fisher as an autosomal dominant disorder with variable penetrance (McKusick, Fisher, 1958). Up to 18 % of all familial IPFs are caused by mutations in the genes of telomerase components: TERT (c.97C>T, c.430G>A, c.1456C>T, c.2240delT, c.2593C>T, c.2594G>A, c.3346_3522del) и TERC (r.37a>g) (Tsakiri et al., 2007). Exome sequencing also made it possible to identify rarer forms of familial IPF caused by mutations in the helicase gene that regulates telomere elongation (RTEL1: c.602delG, c.1451C>T, c.1940C>T, c.2005C>T, c.3371A>C) and in the deadenylation nuclease gene (PARN: IVS4-2a>g, c.529C>T, c.563_564insT, c.751delA, IVS16+1g>a, c.1262A>G) (Stuart et al., 2015). Cases of familial IPF caused by a mutation in exon 5 (+128T>A) in the SFTPC surfactant protein gene are also described (Thomas et al., 2002).

Syndromal IPF develops in autosomal recessive Hermansky– Pudlak syndrome, which is caused by an AP3B1 gene mutation (encodes an intracellular traffic protein). In this case, the specific mutations in the AP3B1 gene are the following: c.1525C>T (p.R509X), c.1739T>G (p.L580R), IVS10+5G>A, IVS11–1G>C (Gochuico et al., 2012).

The promoter region of the mucin gene (MUC5B) contains a highly conserved polymorphic variant rs35705950 for primates, which is associated with sporadic and familial forms of IPF (Seibold et al., 2011). SFTPC gene polymorphisms (G4702C, C4859G, G4877A, G5089A, C5210A, G5236A, G5574A, A5786C, T6108C, C6699T) are associated with sporadic IPF (Lawson et al., 2004). In this form of IPF, shortening of the telomeres of circulating lymphocytes was revealed, which indicates the role of changes in the TERT and TERC genes (Fernandez et al., 2012). According to epidemiological data, familial forms with autosomal dominant inheritance range from 0.5–2 % (in the USA) (Allam, Limper, 2006) to 3.3–3.7 % (in Finland) (Hodgson et al., 2002) of all cases of IPF.

The most reliable data on the genes involved in the pathogenesis of IPF can be obtained from large-scale studies using genome-wide association studies (GWAS). A meta-analysis of five studies of IPF patients compared with healthy controls (the numbers of IPF patients in samples are 88, 61, 54, 22 and 77 from different countries) revealed the haploblock VNTR*2 of the IL1RN gene (encodes an interleukin-1 receptor antagonist), associated with susceptibility to the development of sporadic IPF (Korthagen et al., 2012). In a study of 544 patients with IPF, associations with various alleles of the TOLLIP gene (rs111521887, rs5743894, rs5743890), the SPPL2C gene allele (rs17690703), and the MUC5B gene allele (rs35705950) were found. The TOLLIP gene encodes a Toll-interacting protein involved in the innate immune system; the SPPL2C gene encodes a lysosomal membrane protein with a conserved transmembrane domain (Noth et al., 2013). The role of the MUC5B allelic variant (rs35705950) in the predisposition to IPF was confirmed in a meta-analysis of 2859 patients with IPF (control group consisted of 6901 people) (Lee M.G., Lee Y.H., 2015). The Tollip protein plays an important role in modulating the transport and degradation of TGF-β (Zhu L. et al., 2012). These results are consistent with the role of TGF-β in the pathogenesis of IPF (Chioma, Drake, 2017).

A GWAS conducted in 2016 on 1616 patients (control – 4683 people) showed the association of two haplotypes of the genes of the major histocompatibility complex (HLA): DRB1*15:01 and DQB1*06:02 with the development of IPF. It allowed researchers to suggest the role of autoimmune processes in the development of IPF (Fingerlin et al., 2016). A GWAS conducted in 2020 on DNA samples from 2668 patients showed an association of sporadic IPF with alleles of genes MUC5B (rs35705950), TERC (rs12696304), TERT (rs7725218), DSP (encodes desmoplakin for intercellular contacts, allele rs2076295), ATP11A (encodes a membrane ATPase that regulates calcium ions transport, rs9577395 variant), IVD (encodes isovaleryl-CoA dehydrogenase, rs59424629 polymorphism), AKAP13 (encodes lymphoblastic oncogene, rs62023891 allele), FAM13A (hypoxia inducible gene associated with lung cancer, rs2013701 variant), DPP9 (encodes a serine protease, polymorphism rs12610495) (Allen et al., 2020).

Thus, according to most genetic studies, IPF is associated with allelic variants of the genes responsible for the production of mucin, the functioning of telomeres and the immune system, which indicates a complex pathogenesis of the disease. In addition, IPF is associated with aging. At the molecular level, IPF development involves processes characteristic of aging, including telomere depletion, genomic instability, mitochondrial dysfunction, cellular senescence, and loss of proteostasis (Gulati, Thannickal, 2019). One of the causes of aging is the dysfunction of the immune system and telomeres caused by impaired transposon expression (Mustafin, 2019). This is due to the fact that in evolution, transposons became sources of the nucleotide sequence of both telomeres (Arkhipova et al., 2017) and telomerase encoding genes (Garavis et al., 2013). In Drosophila, the role of telomerase is performed directly by retrotransposons: TAHRE (Telomere Associated and HeT-A Related), TART (Telomere Associated Retrotransposon) и HeT-A (Healing Transposon) (Casacuberta, 2017). In humans, the ability of LINE1 retrotransposons to participate in alternative telomere elongation was revealed (Bondarev, Khavinson, 2016). Transposons likely play a role in the IPF pathogenesis, since familial IPF is most often caused by mutations in the genes maintaining telomeres (the TERC and TERT genes) (Tsakiri et al., 2007; Fernandez et al., 2012), while sporadic forms of IPF are associated with polymorphic variants of these genes (Allen et al., 2020).

Transposons serve as the basis for the epigenetic regulation of ontogenesis (Mustafin, Khusnutdinova, 2019). Transposons are specific genome structures capable of moving to a new locus and occupy 45 % of human DNA. They are classified into DNA transposons (movement by the “cut and paste” mechanism) and retrotransposons (movement with reverse transcription of mRNA and insertion of cDNA) (Wei G. et al., 2016).

## Role of miRNAs in the pathogenesis of idiopathic pulmonary fibrosis

Epigenetic factors include DNA methylation, histone modifications and chromatin remodeling, as well as RNA interference via non-coding RNAs. Transposons are the most important sources of miRNA genes during evolution, in connection with which the MDTE (miRNAs derived from Transposable Elements) database was created in 2016 (Wei G. et al., 2016). Data from this database are taken from the results of the work of various authors (Piriyapongsa et al., 2007; Gu et al., 2009; Filshtein et al., 2012; Tempel et al., 2012; Qin et al., 2015). Investigation of miRNAs can provide information about IPF pathogenesis, as well as become the basis for the development of effective disease therapy. Lung fibroblasts play an important role in the initiation and progression of IPF. Investigation of microRNA expression in these cells revealed a decrease in miR-101 levels in human patients with IPF and in experimental models (bleomycin-induced pulmonary fibrosis) (Huang C. et al., 2017). In the development of IPF, dysregulation of various miRNAs that affect the TGF-β signaling pathways, which induce cell differentiation, migration, invasion, and hyperplastic changes, was revealed. These microRNAs include miR-21, miR-424 (profibrotic); miR-9-5p, miR-18a-5p, miR-26a, miR-27b, miR-101, miR-153, miR-326, miR-489, miR-1343 (antifibrotic) (Kang, 2017).

A pronounced imbalance in the expression of microRNA families miR-29, miR-21-5p, miR-92a-3p, miR-26a-5p, let- 7d-5p in IPF was found, and therefore these molecules are considered as potential therapeutic targets for treatment of the disease (Bagnato et al., 2017). In human lung epithelium with IPF and mice with bleomycin-induced lung fibrosis, a decrease in the level of miR-323a was found, which attenuates TGF-α and TGF-β signaling (Ge et al., 2016). MiR-21 also influences these signaling pathways. The expression of miR-21 is increased in lung tissues of IPF patients and experimental mice. MiR-21 is produced by fibroblasts and regulates Smad7 expression by influencing TGF-β1, promoting extracellular matrix hyperproduction (Liu G. et al., 2010). Low expression of miR-184 in IPF patients correlates with high levels of p63 oncosuppressive protein, knockdown of which reduces TGF-β1-induced lung fibrosis. It was found that miR-184 binds complementarily to the 3′-UTR of the mRNA of the TP63 gene, suppressing its expression (Li J. et al., 2021).

Among the microRNAs listed above associated with IPF (Huang C. et al., 2017), miR-326 (source – hAT-Tip100 DNA transposon) and miR-1343 (source – LINE2 retrotransposon) originated from transposons, according to MDTE and data of various authors (Piriyapongsa et al., 2007; Gu et al., 2009; Filshtein et al., 2012; Tempel et al., 2012; Qin et al., 2015; Wei G. et al., 2016). In 2015, Yang et al. identified significant changes in the levels of 47 different miRNAs in the blood plasma of IPF patients compared with healthy controls (Yang et al., 2015). Of these 47 microRNAs, 4 originated from transposons: miR-31 (from LINE2), miR-302 (from the nonautonomous retroelement SINE/MIR), miR-335 (from SINE/MIR), miR-374 (from LINE2) (Wei G. et al., 2016). These 47 microRNAs are involved in the signaling pathways of TGF-β, mitogen-activated protein kinase (MAPK), PI3K-Akt, Wnt, HIF-1, Jak-STAT, Notch, actin cytoskeleton regulation (Yang et al., 2015). Reduced expression of miR-630 (Li R. et al., 2018) (derived from SINE/ MIR (Wei G. et al., 2016)), miR-708-3p (Liu B. et al., 2018) (from LINE2 (Wei G. et al., 2016)) was detected in the blood plasma of patients with IPF. Elevated levels of transposonderived miRNAs were shown for miR-487b (from SINE/ MIR), miR-493 (from LINE2), miR-495 (from the LTRcontaining retroelement ERVL-MaLT) (Zhang et al., 2021). MiR-340-5p, which promotes fibroblast proliferation in IPF by affecting the ATF and MAPK/p38 pathways (Wei Y.Q. et al., 2020), originated from the TcMar-Mariner DNA transposon (Wei G. et al., 2016).

Table 2 presents data on changes in the expression of miRNAs that originated in evolution from transposons (as well as long non-coding RNAs (lncRNA)) in IPF with a comparative analysis of scientific literature data on these miRNAs in bronchial asthma and chronic obstructive pulmonary disease. As can be seen from Table 2, among 24 miRNAs, 13 of them are unique in the changes in expression in patients with IPF: miR-9-5p, miR-27b, miR-153, miR-184, miR-326, miR-340, miR-374, miR-424, miR-487b, miR-489, miR-493, miR-630, miR-1343. Of these, 8 microRNAs (miR-153, miR-326, miR- 340, miR-374, miR-487b, miR-493, miR-630, miR-1343) are evolutionarily derived from TE (Piriyapongsa et al., 2007; Gu et al., 2009; Filshtein et al., 2012; Tempel et al., 2012; Qin et al., 2015; Wei G. et al., 2016).

**Table 2.**
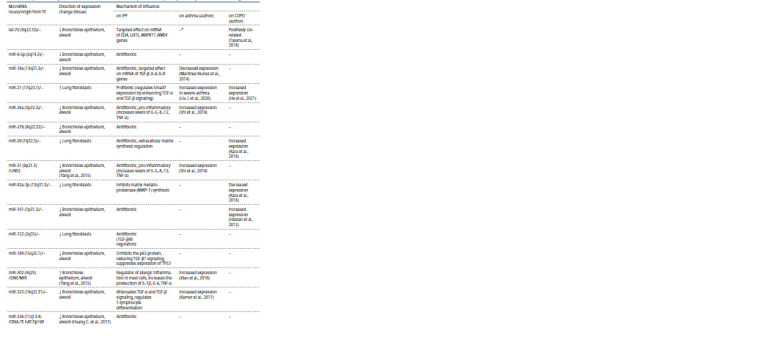
Comparative analysis of the role of microRNAs in the development of idiopathic pulmonary fibrosis and other lung diseases

**Table 3.**
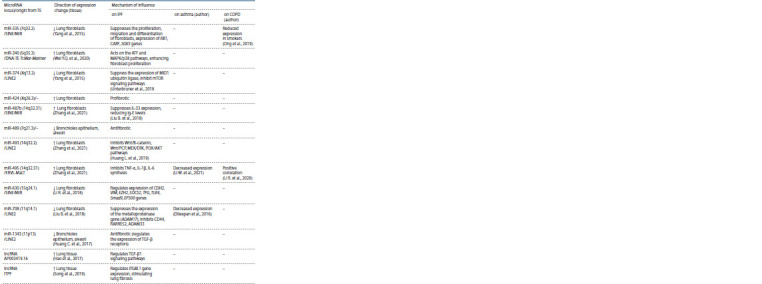
End of Table 2 Note. TE – transposable elements, IPF – idiopathic pulmonary fibrosis, COPD – chronic obstructive pulmonary disease, “–” – no association or correlation data.

Investigation of the role of epigenetic factors in the development of IPF serves as the basis for the development of new methods of targeted therapy for the disease. Potential agents for the treatment of IPF may be non-coding RNAs. It was found that lncRNA PCAT29 (prostate cancer-associated transcript 29), which activates miRNA-221 and suppresses TGF-β, can be used to treat patients with IPF (Liu X. et al., 2018). It was discovered that expression of miR-506, which is complementary to the 3′-UTR of the p65 NF-κB subunit, is downregulated during IPF. Accordingly, the use of miR-506 as a target for targeted therapy may have an impact on apoptosis and inflammation in IPF (Zhu M. et al., 2019). Administration of antisense miR-21 reduced the severity of pathology in mice with bleomycin-induced lung fibrosis, suggesting the potential use of this miRNA in the treatment of IPF (Liu G. et al., 2010). Similar data were obtained for miR-708-3p (Liu B. et al., 2018). Overexpression of miR-184 suppresses TGF-β-induced fibrotic processes in the lung, therefore miR-184 can be considered for targeted therapy of IPF (Li J. et al., 2021). In animal experiments and in clinical studies on patients with IPF, the effectiveness of the interfering sequence for the long non-coding RNA lncITPF (sh-lncITPF), which reduces the index of fibrosis, collagen and vimentin, was also revealed. In patients with IPF, an increased expression of lncRNA-ITPF was revealed, which affects the acetylation of histones H3 and H4 in the promoter region of the ITGBL1 gene, thus stimulating fibrosis. Transcription of lncITPF is under the control of TGF-β1/Smad2/3 (Song et al., 2019). For IPF treatment, the DR8 peptide (DHNNPQIR-NH2), which has a powerful antioxidant activity, was proposed. In an animal experiment with bleomycin-induced IPF, it was shown that after the use of DR8, fibrosis indicators, including profibrogenic and pro-inflammatory cytokines and marker proteins, were significantly reduced. DR8 reduced pathological changes caused by bleomycin, as well as collagen deposits (especially COL1). In vivo experiments showed that DR8 is able to suppress the proliferation and generation of reactive oxygen species stimulated by TGF-β1 (Wang et al., 2019).

Long non-coding RNAs (lncRNAs) are epigenetic factors, since they have transcriptional, post-transcriptional, and translational regulatory effects on the functioning of the genome. This effect is realized both due to the secondary structure of RNA, which provides interaction with proteins, and through hybridization with DNA and RNA due to the complementarity of nucleotides. Many lncRNA genes evolved from transposons (Johnson, Guigo, 2014). According to the NONCODEv4 database (http://www.noncode.org), more than 96,000 lncRNA genes have been annotated in humans, many of which contain TE sequences, which indicates the role of TEs in the origin of lncRNA genes (Johnson, Guigo, 2014). In addition, lncRNA can be formed during the processing of transcripts of LTRcontaining retroelements (Lu et al., 2014) or LINE retrotransposons (Honson, Macfarlan, 2018). Analysis of GENOCODE and expressed RNA sequences showed that the majority of lncRNAs originated from transposons, since at least 83 % of them contain one or more retroelement fragments. On average, about 41 % of all lncRNA nucleotide sequences are identical to transposons (Kelley, Rinn, 2012). Thus, changes in lncRNA expression during IPF could indicate the role of transposons in the pathogenesis of the disease. Indeed, in 2017, Hao et al.determined a decrease in the levels of 1,376 different lncRNAs and an increase in the levels of 440 lncRNAs in the blood plasma of patients with ILF compared with healthy controls. The highest level was observed for lncRNA AP003419.16, which is involved in TGF-β1 signaling pathways and can be used as a marker of disease (Hao et al., 2017). 

## Influence of transposons on pulmonary fibrosis pathogenesis

The above data indicate the role of transposons in the emergence of noncoding RNAs that are involved in the pathogenesis of IPF and many other human diseases. The obtained results of molecular genetic studies of IPF are consistent with this assumption. It refers to the influence of transposons on the aging processes that are involved in the pathogenesis of IPF and other multifactorial diseases (Gulati, Thannickal, 2019). In aging, retrotransposons containing long terminal repeats (Navalainen et al., 2018) and LINE1 (Mahmood et al., 2020) are activated. Moreover, their overexpression during aging enhances the production of interferon, contributing to aseptic inflammation in tissues (De Cecco et al., 2013).

Transposons (due to the relationship with microRNAs derived from them) are involved in the functioning of the immune system, the changes in which are associated with IPF (Korthagen et al., 2012; Noth et al., 2013; Fingerlin et al., 2016). For example, the miR-31 microRNA derived from LINE2 has a pro-inflammatory effect, enhancing the synthesis of IL-5,-8,-12, TNF-α (Shi et al., 2019); miR-302 (evolved from SINE/MIR) increases production of IL-1β, IL-6, TNF-α (Xiao et al., 2018). SINE/MIR are also a source of miR-487b, which represses IL-33 expression, reducing Ig-E levels (Liu H.C. et al., 2018). MiR-495 derived from ERVL-MaLT inhibits the synthesis of TNF-α, IL-1β, IL-6 (Li W. et al., 2021). In mammalian evolution, RAG genes were domesticated from ancient DNA transposons for V(D)J recombination in the immune system. Vertebrate antigen-specific immunity has two main features of DNA transposons. The components of immunity consist of recombinase (encoded by the RAG1 and RAG2 genes) and mobile DNA (limited to specific sites that the recombinase recognizes). RAG proteins are homologous to Tc1-element transposase (Lescale, Deriano, 2016). LTR-containing retroelements are involved in the regulation of the human immune system, as they are enhancers for the HLA-G gene (Chuong, 2018).

Transposons also affect the sirtuins (Wyman et al., 2017) and TGF-β (Liu G. et al., 2010; Chioma, Drake, 2017; Kang, 2017) involved in the pathogenesis of IPF. SIRT7 epigenetically represses LINE1 expression throughout the genome. An important role in this process is played by the interaction of SIRT7 with lamins A/C, since SIRT7 ensures the deacetylation of histone H3K18, facilitating the interaction of LINE1 with the nuclear lamina (Vazquez et al., 2019). Derived from an LTR-containing retroelement, the PEG10 gene encodes a PEG10-RF1 protein that interacts with members of the TGF-β type I and II superfamily (Lux et al., 2005). The role of evolutionarily young retroelements in the regulation of TGF-β pathways, along with PDGF, EGFR and p38 signaling, was revealed (Nikitin et al., 2018). The role of retroelements in the epithelial-mesenchymal transition important for the development of IPF was shown (Sgalla et al., 2018; Li J. et al., 2021), which is induced by the non-autonomous retrotransposon Alu due to the modulating of miR-566 expression (Ruocco et al., 2018). Telomere dysfunction leading to the development of IPF (Mathai et al., 2015; Chioma, Drake, 2017; Allen et al., 2020) and other diseases is likely associated with changes in the activity of transposons, which are the evolutionary sources of genes involved in the functioning of telomeres (Arkhipova, 2017) and the telomerase gene (Garavis et al., 2013).

## Conclusion

The investigation of epigenetic factors in the development of IPF is a promising direction in revealing the pathogenesis of the disease and developing more effective methods of its therapy. Through the study of miRNAs, it was shown that IPF is associated with an imbalance in the epigenetic regulation of the genome. Therefore, the reason for the development of IPF may be an imbalance in the control of the work of the genome by dynamic structures that play a role in age-associated pathology and aging of the body. The most appropriate control elements are transposons, since they affect the functioning of the immune system and are closely related to it evolutionarily. It has been suggested that the study of the role of transposons in the pathogenesis of IPF can reveal the pathways of the molecular cascade of the disease. Evidence for the role of transposons in the pathogenesis of IPF is the evolutionary emergence of long noncoding RNAs and miRNAs from transposons. Analysis of the MDTE database and scientific literature revealed 12 specific IPF-associated miRNAs that originated from transposons. Eight of these 12 microRNAs (miR-153, miR-326, miR-340, miR-374, miR-487b, miR-493, miR-630, miR-1343) are unique, since the change in their expression is specific for IPF and has not been described with other diseases of the bronchopulmonary system.

## Conflict of interest

The authors declare no conflict of interest.
